# Coronavirus disease 2019 vaccination and menstrual cycle changes: A cross-sectional study on females of reproductive age in Saudi Arabia

**DOI:** 10.1097/MD.0000000000041656

**Published:** 2025-02-21

**Authors:** Mamdoh Eskandar, Alshaima Alassim, Fatima Riaz, Syed Esam Mahmood, Nouf Khaled Alshehri, Ahmed Ali Al Asim, Mohamed Almodeer, Ausaf Ahmad

**Affiliations:** a Department of Obstetrics and Gynecology, College of Medicine, King Khalid University, Abha, Saudi Arabia; b College of Medicine, King Khalid University, Abha, Saudi Arabia; c Department of Family and Community Medicine, College of Medicine, King Khalid University, Abha, Saudi Arabia; d Department of Medicine, Armed Forces Hospital South Region, Khamis Mushait, Saudi Arabia; e Khamees Mushait Military Hospital, Khamis Mushait, Saudi Arabia; f Department of Community Medicine, Kalyan Singh Government Medical College, Bulandshahr, India.

**Keywords:** COVID-19, menstrual cycle, reproductive age, vaccination

## Abstract

Data supporting the evidence of changes in the menstrual cycle and premenstrual symptoms associated with coronavirus disease (COVID-19) is quite scarce. To determine the association between COVID-19 vaccination and menstrual cycle changes and its relationship with different types of vaccines among women of reproductive age in Abha City, Saudi Arabia. A cross-sectional study was conducted from January 2022 to June 2022, among participants from Asser region of Saudi Arabia by using a self-administered questionnaire through an electronic survey. Data analysis was performed using SPSS version 16.0. Among 1208 study subjects, majority (66.9%) of females had menarche at the age of <13 years, 17.2% had irregular periods, and 24.8% reported an average regularity of periods (23–35 days). A statistically significant association was found among females who experienced a change in their menstrual cycle after receiving COVID-19 vaccine. Mood swings and lower back pain were common symptoms of premenstrual syndrome symptoms. Only 15% females reported a delay in conception. Out of 176 females, 40% showed 6 month delay in conception after receiving vaccine. Multivariate logistic regression analysis showed that age, regularity of periods, and usual volume of bleeding were significantly associated with changes in the menstrual cycle after vaccination. The relationship between COVID-19 vaccine and associated changes on the menstrual cycle and premenstrual syndrome was established in our study. Further research is needed to produce concrete evidence regarding its relationship to eliminate vaccine hesitancy among women.

## 1. Introduction

The world has faced a severe sinister virus named severe acute respiratory syndrome coronavirus 2 (SARS-CoV-2), which was discovered during the outbreak of a highly transmissible respiratory disease in Wuhan, a city in Republic of China, in 2019. This outbreak has become a pandemic, known as coronavirus disease 2019 (COVID-19), which continues to spread worldwide.^[1]^ COVID-19 pandemic has been a hot topic for 3 years, and many studies have highlighted its effect on different aspects of life. SARS-CoV-2 infection caused many psychological problems like depression and anxiety along with major effects on the respiratory, cardiovascular, neurological, and musculoskeletal systems.^[2]^ Multiple complications occurred not only as a result of the disease itself but also due to different treatment modalities given initially to manage COVID-19 cases.^[3]^ Further, the effect of COVID-19 vaccine has been under focus since its development and administration.^[4]^ Rapid development of COVID-19 vaccine and its compulsory administration to the entire population by defining risks versus benefits was a critical regulatory medical decision, although vaccine safety measures were considered.^[5]^ However, COVID-19 vaccine is of great concern in general population, and they have various opinions and perceptions regarding the side effects associated with the vaccine, causing anxiety among common people and avoidance behavior toward receiving the vaccine among adults and children as well as the elderly population.^[6]^ Vaccine hesitancy, a multifactorial phenomenon caused by the complex interaction of different social, cultural, political, and personal factors, is a common issue worldwide.^[7]^ Multiple attitudes were encountered regarding COVID-19 vaccine administration, including uncertainty and hesitation regarding the newly invented vaccine.^[8]^ Different groups of people have different perceptions of the vaccine.^[9]^ Some individuals experienced short-term side effects such as fatigue, fever, body ache, nausea, vomiting, headache, joint pain, and joint swelling after receiving the vaccine.^[10]^ Some studies have reported vaccine-associated myocardial infarction^[11]^; acute peripheral facial paralysis (Bell palsy); facial swelling; swelling of the lips, face, or tongue associated with anaphylaxis^[12]^; vaccine-induced prothrombotic immune thrombocytopenia^[13]^; and severe scleroderma.^[14]^ Moreover, few people believe that COVID-19 vaccine affects fertility and causes menstrual problems; however, little is known about its impact on female reproductive system and menstrual cycle. Studies are insufficient to determine the impact of COVID-19 vaccination on menstrual cycle.^[15]^ Nevertheless some viral infections such as human immunodeficiency virus, hepatitis B, and hepatitis C virus infection have been reported to correlate with complications in the reproductive system and menstrual cycle changes; it is suggested that vaccines for prevention of viral infections could be associated with menstrual changes as well. These viral infections can cause amenorrhea, menorrhagia, prolonged and heavy menstruation, scanty and low flow, various symptoms related to premenstrual syndrome, absence of menstruation, and dysmenorrhea.^[16]^ Some studies have also reported menstrual and premenstrual changes associated with COVID-19 infection as well as with the vaccine, but data to support this evidence are quite scarce. We aimed to study changes in the menstrual cycle because it reflects the general health status of women. Irregularities in the menstrual cycle pose a greater risk for the development of metabolic syndrome, dyslipidemia, and infertility.^[17]^

Considering the above facts, the study was aimed to determine the association of COVID-19 vaccination with menstrual cycle changes and its relationship to different types of vaccines among women of reproductive age in Abha City of Saudi Arabia, which will help clinicians to provide better care and services to patients. We hypothesized that menstrual cycle changes could be found among women of reproductive age who have received COVID-19 vaccine.

## 2. Methods

### 2.1. Study design, population, and sample

This cross-sectional study focused on female participants of reproductive age residing in Abha City, which is located in the Aseer Province of Saudi Arabia. The study was conducted over a 6-month period, specifically from January 2022 to June 2022. The reference population for this research included all females within the reproductive age range living in Abha City during that time. This means that the study aimed to gather data and insights from a diverse group of women in that specific region, providing a comprehensive understanding of their reproductive health and COVID-19 vaccination status. This sample would consist of a selected number of females of reproductive age who participated in the research, providing data on their COVID-19 vaccination status and any changes they experienced in their menstrual cycles. This group specifically includes those who agreed to participate in the study and had received the COVID-19 vaccine. The study aimed to gather insights from this demographic during the study period. To ensure the reliability of the data, certain individuals were excluded from the study. This included females who did not consent to participate, those who were experiencing menopause, pregnancy, or lactation, and those with irregular menstrual cycles due to gynecological issues. Additionally, any participants who had changes in their menstrual cycle at the time of data collection or prior to receiving the vaccine were also excluded. In total, 1208 participants were selected using snowball sampling, which helped to reach a broader network of eligible individuals within the target population.

### 2.2. Sample size calculation

Assuming the maximum variability, which is equal to 50% (*P* = .5) and taking 95% confidence level (*Z* score = 1.96) with ±3% precision (d), 15% nonresponse, the formula for required sample size is, $n=z^{2}\;p\left(1-p\right)/d^{2}$. The required sample size was calculated to be 1067, assuming a 15% nonresponse rate. To account for this, a total of 1227 participants were approached for data collection. After data collection, 1208 participants’ data were retained for final analysis, as 19 participants’ data were excluded due to incomplete or inaccurate information.

### 2.3. Procedure

The survey design process involved several key steps, including defining the objectives, developing questions, and ensuring clarity and relevance. A self-structured questionnaire was designed by a team of principal authors and coauthors after a thorough literature search based on observations during clinical practice.^[18]^ All information was gathered by the principal investigator and coinvestigators using an electronic survey self-structured questionnaire after obtaining informed consent from the participants. It consisted of close-ended questions designed through the SurveyMonkey application and distributed through personal contacts with family, friends, colleagues, and by using different social media resources such as Twitter, WhatsApp, and Instagram. The questionnaire was translated from English to Arabic (local language) by a bilingual person to enable easy understanding of the questions and focusing on cultural appropriateness to avoid questionnaire bias. When it came to translating the survey into Arabic, it is important to focus on accurate language translation while also considering cultural nuances to ensure that the questions are understood correctly by Arabic-speaking respondents. This process involved collaboration with a bilingual expert to maintain the integrity of the survey’s intent and meaning. Before administering the final version of the questionnaire, a pretest was performed on randomly selected 25 females in the region to ensure the questionnaire’s validity, reliability, applicability, and average filling time. The overall reliability coefficient (α-Cronbach) was 0.76. A 17-item questionnaire was constructed with multiple parts, including sociodemographic information, gynecological characteristics, pattern of menstrual bleeding, cycle change after COVID-19 vaccination, changes in symptoms of premenstrual syndrome, and delayed conception. Based on the definition of infertility, subjects who did not conceive within 1 year were considered to have a delay in conception.

### 2.4. Ethical considerations

This study was approved by the Ethics Committee of King Khalid University (ECM# -613 KKU). Informed consent was obtained from each subject, after receiving approval of the protocol by institutional review board. Participants were assured that their identities would remain anonymous and unidentified throughout the study, and the data would be utilized only for research purposes by keeping their identities confidential.

### 2.5. Statistical analyses

Analyses were performed using SPSS 16.0 version (SPSS Inc., Chicago, IL). The results are presented as frequency and percentage graphs, such as bar diagrams. Baseline characteristics were compared using the chi-squared test for categorical variables. Statistical significance was set at *P* < .05. The imputation method was used to handle the missing data. To examine the cross-sectional association among gynecological characteristics, demographic factors, and changes in the menstrual cycle after receiving COVID-19 vaccine, we fitted a multiple regression analysis model with any change in the cycle after receiving the vaccine as an independent variable to estimate odds ratio (OR) and 95% confidence interval (CI) of categorized cycle characteristics.

## 3. Results

Among 1208 study subjects included in the present study, 1058 (87.58%) were Saudi participants. Approximately 848 (70.19%) females were aged <30 years. General characteristics of the study participants, such as nationality, age, marital status, and contraceptive use, were evaluated. The association of characteristics and changes in cycle after receiving the vaccine, indicating that the majority of females 809/1208 had menarche at the age of <13 years. The number of study participants who experienced changes in the cycles after receiving the vaccine was 737 (61.0%). A higher proportion of married females self-reported a change in the cycle after receiving the vaccine than unmarried women, but the difference was not statistically significant (Table 1).

**Table 1:** Association of sociodemographic and gynecological characteristics with the changes in menstrual cycle after receiving COVID-19 vaccine.

Characteristics		Any change in the cycle after receiving the vaccine?		Total	*P*-value
		No (n = 471)	Yes (n = 737)		
Nationality	Non-Saudi	68	82	150	.088
		45.3%	54.7%	100.0%	
	Saudi	403	655	1058	
		38.1%	61.9%	100.0%	
Age (years)	15–29	340	508	848	.474
		40.1%	59.9%	100.0%	
	30–39	88	156	244	
		36.1%	63.9%	100.0%	
	40–49	43	73	116	
		37.1%	62.9%	100.0%	
Age of menarche	≤13 yr	334	475	809	.019
		41.3%	58.7%	100.0%	
	≥14 yr	137	262	399	
		34.33%	65.66%	100.0%	
Marital status	Unmarried	323	516	839	.597
		38.5%	61.5%	100.0%	
	Married	148	221	369	
		37.6%	62.4%	100.0%	
Ever being pregnant	No	366	549	915	.203
		40.0%	60.0%	100.0%	
	Yes	105	188	293	
		35.8%	64.2%	100.0%	
History of chronic disease	Yes	58	101	159	.485
		36.5	63.5	100.0	
	No	413	636	1049	
		39.4%	60.6%	100.0%	
Type of contraceptive currently using	Currently not using	396	638	1034	.387
		38.3%	61.7%	100.0%	
	Implant	3	10	13	
		23.1%	76.9%	100.0%	
	Intrauterine device	17	19	36	
		47.2%	52.8%	100.0%	
	Pills	42	51	93	
		45.2%	54.8%	100.0%	
	Others	13	19	32	
		40.6%	59.4%	100.0%	

About 300/1208 (24.8%) females reported that the average regularity of periods was usually 23 to 35 days and 208/1208 (17.2%) had irregular periods. During a typical menstrual period, most females 445/1208 reported 3 to 7 days of bleeding, whereas 93/1208 (7.6%) females had heavy bleeding during menstrual flow, in which few of them (0.9%, 12/1208) reported soaking of 7 to 9 times a tampon or pad with 4-h interval. It also shows the association between COVID-19 vaccine dose taken by female study subjects and changes in the menstruation cycle after receiving the vaccine. After the first, second, third, and fourth doses of the vaccine, and type of vaccine taken by female subjects showed a significant association with those who experienced a change in cycle after receiving the vaccine (Table 2).

**Table 2:** Association of change in menstrual cycle after receiving COVID-19 vaccine according to menstrual bleeding pattern.

Change in menstrual cycle and bleeding pattern after receiving COVID-19 vaccine					
Characteristics		Any change in the cycle after receiving the vaccine?		Total (N = 1208)	*P*-value
		No (n = 471)	Yes (n = 737)		
Regularity of periods	Regular (23–35 d)	142	158	300	.000
		47.3%	52.7%	100.0%	
	Irregular (>35 d or < 23 d)	329	579	908	
		36.2%	63.8%	100.0%	
Number of days of bleeding during a usual menstrual period	<3	50	76	126	.417
		39.7%	60.3%	100.0%	
	3–7	183	262	445	
		41.1%	58.9%	100.0%	
	>8	16	37	53	
		30.2%	69.8%	100.0%	
	Did not answer	222	362	584	
		38.0%	62.0%	100.0%	
Usual volume of bleeding	Heavy	22	71	93	.005
		23.7%	76.3%	100.0%	
	Light	30	36	66	
		45.5%	54.5%	100.0%	
	Normal	197	268	465	
		42.4%	57.6%	100.0%	
	Did not answer	222	362	584	
		38.0%	62.0%	100.0%	
Number of times would you soak a tampon or pad in 4 h	<3	192	283	475	.847
		40.4%	59.6%	100.0%	
	4–6	53	84	137	
		38.7%	61.3%	100.0%	
	7–9	4	8	12	
		33.3%	66.7%	100.0%	
	≥10	222	362	584	
		38.0%	62.0%	100.0%	

COVID-19 vaccine dose and change in menstrual cycle after receiving COVID-19 vaccine					
Characteristics		Any change in the cycle after receiving the vaccine?		Total	*P*-value
		No (n = 471)	Yes (n = 737)		
Cycle change experienced after which dose of vaccine	First	12	265	277	.000
		4.3%	95.7%	100.0%	
	Second	11	317	328	
		3.4%	96.6%	100.0%	
	Third	4	140	144	
		2.8%	97.2%	100.0%	
	Fourth	0	4	4	
		.0%	100.0%	100.0%	
	Do not remember	444	11	455	
		97.6%	2.4%	100.0%	
Type of vaccine responsible for cycle change	AstraZeneca (Vaxzevria, Covishield)	10	160	170	.000
		5.9%	94.1%	100.0%	
	Pfizer-BioNTech (Comirnaty)	20	527	547	
		3.7%	96.3%	100.0%	
	Moderna (Spikevax)	7	41	48	
		14.6%	85.4%	100.0%	
	Do not remember	434	9	443	
		98.0%	2.0%	100.0%	

**Figure 1.**
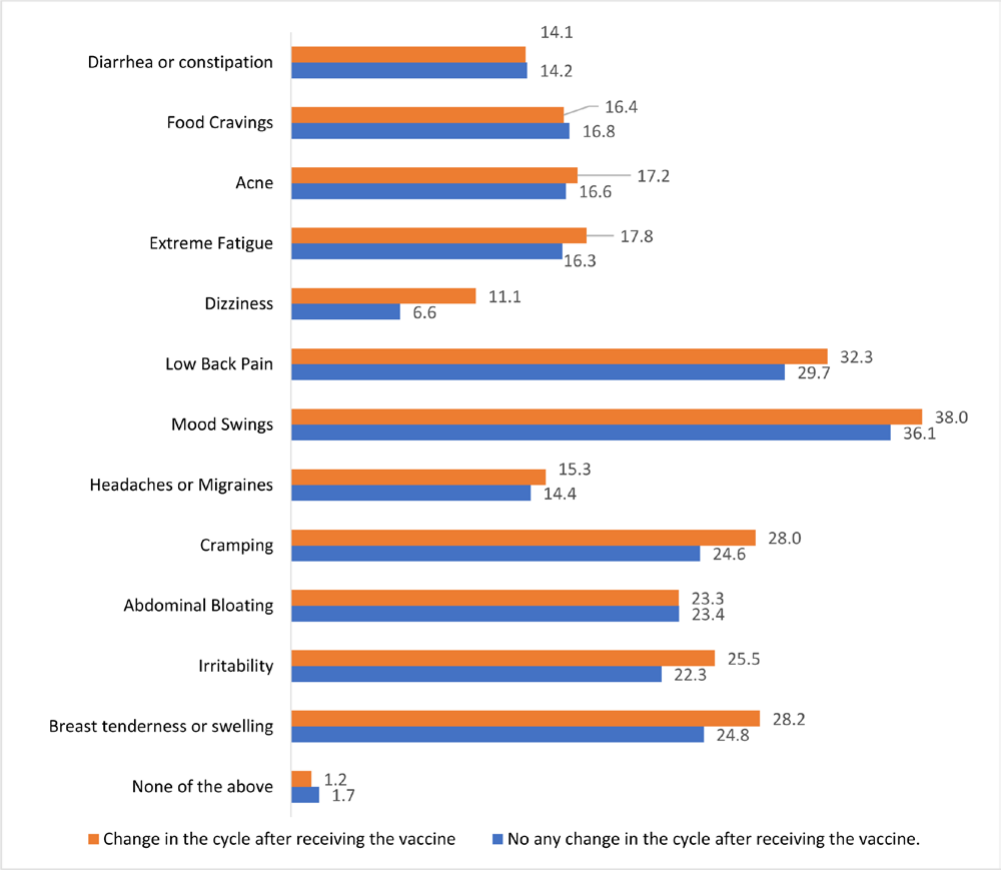
Changes in the symptoms of premenstrual syndrome (PMS) after COVID-19 vaccination.

**Figure 2.**
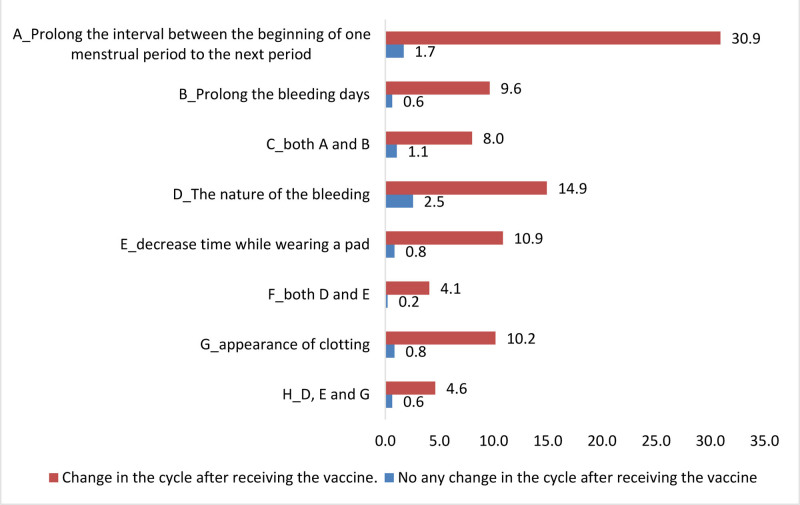
Change versus no change in the menstrual cycle after receiving COVID-19 vaccine.

Figure 1 shows a comparison of female study subjects with and without changes in premenstrual syndrome (PMS) after receiving the vaccine. Mood swings and lower back pain are the common symptoms of PMS. Figure 2 illustrates the prolonged interval between the beginning of 1 menstrual period and the next, which best describes changes in the menstrual cycle.

Among females who were infected with COVID-19 virus, 52.9% experienced no change in the cycle after receiving the vaccine. Most female study participants were infected before vaccination.

The percentage distribution of study subjects who showed a delay in conception after receiving the vaccine and the duration of delay in conception. Among all participants, only 176 (15%) females showed a delay in conception. Of 176 females, the majority 70 (40%) showed 6 month delay in conception after receiving the vaccine (Table 3).

**Table 3:** Frequency distribution of delay in conception.

Characteristics	Categories	Number	Percentage (%)
Any delay in conception after receiving the vaccine? (n = 1208)	No	1032	85.43
	Yes	176	14.57
If yes, then how long was the delay in conception?	<6 mo	70	39.77
	>6 mo	24	13.64
	1 yr	38	21.59
	2 yr	44	25.00

The results of multivariate logistic regression analysis, which revealed that cycle change experienced after the fourth dose, Moderna (Spikevax) (OR: 2.36, 95% CI: 0.12–46.29 and OR: 2.71, 95% CI: 0.98–7.61), usual volume of bleeding, light and normal (OR: 2.68, 95% CI: 1.36–5.31 and OR: 2.37, 95% CI: 1.42–3.96) were found to be significantly associated with changes in the cycle after receiving the vaccine.

On the contrary, cycle change experienced after the second and third dose of the vaccine (OR: 0.76, 95% CI: 0.33–1.76 and OR: 0.63, 95% CI: 0.19–1.99) and Pfizer BioNTech (Comirnaty) (OR: 0.60, 95% CI: 0.27–1.32) were not found to be associated with changes in the cycle after receiving the vaccine (Table 4).

**Table 4:** Multivariate logistic regression analysis of parameters contributing to change in menstrual cycle after receiving COVID-19 vaccine.

Characteristics	Category	OR	95% CI		Any change in the cycle after receiving the vaccine?		Total	*P* value
			Lower limit	Upper limit	Yes (n = 737)	No (n = 471)		
Nationality	Non-Saudi	Ref.			82	68	150	.088746
	Saudi	0.74	0.52	1.04	655	403	1058	
Age of menarche	≤13 yr	Ref.			475	334	809	.019846
	≥14 yr	0.74	0.57	0.95	262	137	399	
Regularity of periods	Irregular (>35 d or < 23 d)				329	579	908	
	Regular	0.51	0.39	0.66	158	142	300	.00001
Usual volume of bleeding	Heavy	Ref.			71	22	93	
	Light	2.68	1.36	5.31	36	30	66	.003889
	Normal	2.37	1.42	3.96	268	197	465	.000743
	Did not answer	1.97	1.19	3.28	362	222	584	.007395
Cycle change experienced after which dose of vaccine	First	Ref.			265	12	277	
	Second	0.76	0.33	1.76	317	11	328	.513174
	Third	0.63	0.19	1.99	140	4	144	.417307
	Fourth	2.36	0.12	46.29	4	0	4	.57
	Do not remember	891.36	387.83	2048.63	11	444	455	.00001
Type of vaccine responsible for cycle change	AstraZeneca (Vaxzevria, Covishield)	Ref.			160	10	170	
	Pfizer BioNTech (Comirnaty)	0.60	0.27	1.32	527	20	547	.205472
	Moderna (Spikevax)	2.71	0.98	7.61	41	7	48	.047115
	Do not remember	771.55	307.90	31933.39	9	434	443	.00001

## 4. Discussion

This study explored the association between COVID-19 vaccine and changes in menstrual cycle. However, definitive evidence regarding the mechanism underlying the association between COVID-19 vaccine and menstrual cycle changes is still not conclusive, and there is a lot of room to understand its short-term effect on menstruation. In this study, we attempted to determine the association between COVID-19 vaccine and changes in the menstrual cycle and symptoms of PMS. The majority of the respondents in our study were Saudi nationals. In this study, approximately 62.4% of married women experienced menstrual problems after receiving COVID-19 vaccine, and approximately 61% of the study participants reported changes in their menstrual cycle. Another study showed similar result (66.6%) for menstrual disturbance among women administered COVID-19 vaccine.^[19]^ However, other studies reported variable percentages (22%–70.6%) of menstrual disturbances.^[20]^ Our study results are comparable with those of a Lebanese study that reported significant associations between irregular cycles and marital status (OR 2.18) and menarcheal age (OR 4.76).^[21]^

In this study, women who observed changes in their menstrual cycle after having been vaccinated for COVID-19 were mainly aged between 30 and 40 years (63.9%), married and had been pregnant (64.2%). This is comparable to another study, where 48.3% of the study population was aged 30–40 years, which is markedly lower than that of the present study; 74.8% were in a relationship, which is slightly higher than that in the present study; only 10.2% were parous women, which is markedly lower than that of the present study; and 55.2% received vaccine manufactured by Pfizer, which is also considerably lower than that of the present study.^[21]^

However, a study conducted in the United Kingdom suggested that the vaccine brand is not associated with differences in the timing or flow of the next menstrual period.^[22]^ This is similar to the results of our study, as multivariate logistic regression did not show any association between vaccine by Pfizer and menstrual cycle changes.

Although the literature provides some evidence regarding the relationship between menstrual changes reported after both mRNA and adenovirus vector COVID-19 vaccines, which is likely to be a result of immune response to vaccination rather than a specific vaccine component.^[23]^ Age at menarche in the study population was above 14 years in 65.66% of females, menstrual flow was irregular among 74.5% of respondents, usual menstrual cycle was more than 8 days among 69.8% females, and menstrual flow was heavy among 76.3% of those who have observed a change in menstruation after vaccination in our study. However, another population-based study conducted in Jordan showed that the age at menarche was below 15 years in 59.8% of the study population, which is nearly equal to our study population; menstrual flow decreased only in 15.5% of the population, which is lower than that reported in the present study; menstrual flow duration was 8–10 days in 12.1% of females, which was significantly lower than that in the present study; and menstrual flow was heavy in 24.5% of the participants, which was significantly lower than our study results.^[24]^ This could be attributed to the fact that SARS-CoV-2 infection and COVID-19 can affect the hypothalamic–pituitary–ovarian–endometrial axis, resulting in changes in the menstrual cycle. Hypothalamic hypogonadism may occur in the presence of any severe illness, including COVID-19, resulting in temporary amenorrhea or infrequent menses. This may explain the relationship between COVID-19 vaccination and menstrual changes.^[25]^ Other studies have also suggested an association of flu and human papillomavirus vaccines with menstrual changes; however, the mechanism by which the vaccine affects menstrual cycle remains under-researched and undetermined.^[26]^

It is evident that cytokine production as a result of immune response either from the vaccine or the infection itself may transiently interfere with the hypothalamic–pituitary–ovarian axis; thus, ovarian hormones that drive the menstrual cycle are transiently disturbed.^[27]^

Approximately 95.7% of the study participants who have experienced changes in their cycle after COVID-19 vaccination received 1 dose, 96.6% received 2 doses, 97.2% received 3 doses, and 100% received 4 doses. Laganà et al reported that approximately 50% to 60% of women of reproductive age who received the first dose of COVID-19 vaccine experienced menstrual cycle irregularities, regardless of the type of vaccine administered, with a slightly higher (60%–70%) occurrence after consecutive doses.^[26]^ The majority of our study participants did not use any type of contraceptive; however, those who were using oral pills had more changes in their menstrual cycle. A study conducted in the UK suggested that participants on progesterone-only contraception were likely to report significantly heavier flow of their postvaccination period than usual, compared to participants on combined or no hormonal contraception. Approximately 15% of our study participants experienced delay in conception, of which 40% delayed conception for 6 month period, whereas 18.5% of participants experienced trouble conceiving, consistent with our study results.^[27]^ One possible explanation is that innate immune cells transiently interfere with reproductive hormones, subsequently causing prolonged cycles; hence, delay in conception is theoretically possible.^[28]^ However, there is no evidence confirming the relationship between COVID-19 vaccine and infertility. Approximately 11.4% had increased menstrual cramps in a study by Lagana et al.^[26]^ Changes in premenstrual symptoms have been reported among 1.7% of participants receiving the first dose and 1.3% receiving the second dose.^[29]^ Moreover, approximately 30.9% of our study participants showed a prolonged interval from 1 menstrual period to the next period, which is also supported by Alghamdi AN et al who reported several menstrual abnormalities following COVID-19 vaccination, including increased cycle duration, pain, and bleeding.^[30]^

This study had some limitations. The snowball sampling method was useful but suffered limitation with potential bias. The study sample cannot be generalized to women of reproductive age in the entire Kingdom of Saudi Arabia, because the questionnaire was used online among participants belonging to Abha City. Factors such as body mass index, depression, and anxiety, which may have affected the menstrual cycle during the lockdown period, were not addressed in our study. Subjective responses were presented using descriptive statistics that cannot be generalized to other populations.

The study involving 1208 subjects reveals several important clinical implications regarding menstrual and reproductive health. Notably, a significant majority of females (66.9%) experienced menarche before the age of 13, highlighting the need for enhanced awareness and education on menstrual health in younger populations to address potential health issues early. Additionally, many females reported changes in their menstrual cycles after receiving the COVID-19 vaccine, emphasizing the necessity for healthcare providers to monitor menstrual health as part of postvaccination care and to address any concerns related to irregularities. The study also found that factors such as age, regularity of periods, and bleeding volume should be considered when evaluating menstrual changes postvaccination. Common premenstrual symptoms like mood swings and lower back pain may require further investigation due to their impact on quality of life. Furthermore, the finding that 40% of females experienced a 6-month delay in conception after vaccination points to the need for additional research into the reproductive health implications of COVID-19 vaccines. Overall, these results stress the importance of ongoing research and effective communication between healthcare providers and patients regarding menstrual and reproductive health following vaccination.

## 5. Conclusion

The relationship between COVID-19 vaccine and the associated changes in the menstrual cycle and premenstrual syndrome was established in our study. Further research is needed to produce concrete evidence about its relationship to eliminate vaccine hesitancy and acceptability among women of reproductive age. Further studies should investigate factors such as weight gain, depression, and anxiety, which may be attributed to the changes in the menstrual cycle.

## Acknowledgments

We would like to thank Editage (www.editage.com) for English language editing.

## Author contributions

**Conceptualization:** Mamdoh Eskandar.

**Data curation:** Mamdoh Eskandar, Ausaf Ahmad.

**Formal analysis:** Ausaf Ahmad.

**Investigation:** Mamdoh Eskandar, Alshaima Alassim, Nouf Khaled Alshehri, Ahmed Ali Al Asim, Mohamed Almodeer.

**Methodology:** Mamdoh Eskandar.

**Project administration:** Mamdoh Eskandar.

**Resources:** Mamdoh Eskandar.

**Software:** Ausaf Ahmad.

**Supervision:** Mamdoh Eskandar.

**Validation:** Mamdoh Eskandar.

**Visualization:** Mamdoh Eskandar.

**Writing – original draft:** Fatima Riaz, Syed Esam Mahmood.

**Writing – review & editing:** Fatima Riaz, Syed Esam Mahmood.

## References

[1] Cucinotta D Vanelli M. WHO declares COVID-19 a pandemic. Acta Biomed. 2020;91:157–60. 32191675 10.23750/abm.v91i1.9397 PMC7569573

[2] Douglas M Katikireddi SV Taulbut M McKee M McCartney G. Mitigating the wider health effects of Covid-19 pandemic response. BMJ. 2020;369:m1557. 32341002 10.1136/bmj.m1557 PMC7184317

[3] Sørvoll IH Horvei KD Ernstsen SL. An observational study to identify the prevalence of thrombocytopenia and anti-PF4/polyanion antibodies in Norwegian health care workers after COVID-19 vaccination. J Thromb Haemost. 2021;19:1813–8. 33909350 10.1111/jth.15352 PMC8237070

[4] Mathioudakis AG Ghrew M Ustianowski A. Self-reported real-world safety and reactogenicity of COVID-19 vaccines: a vaccine recipient survey. Life (Basel). 2021;11:249. 33803014 10.3390/life11030249 PMC8002738

[5] Graham BS. Rapid COVID-19 vaccine development. Science. 2020;368:945–6. 32385100 10.1126/science.abb8923

[6] Peeples L. News feature: avoiding pitfalls in the pursuit of a COVID-19 vaccine. Proc Natl Acad Sci USA. 2020;117:8218–21. 32229574 10.1073/pnas.2005456117 PMC7165470

[7] Rief W. Fear of adverse effects and COVID-19 vaccine hesitancy: recommendations of the treatment expectation expert group. JAMA Health Forum. 2021;2:e210804. 36218819 10.1001/jamahealthforum.2021.0804

[8] Opel DJ Mangione-Smith R Taylor JA. Development of a survey to identify vaccine-hesitant parents: the parent attitudes about childhood vaccines survey. Hum Vaccin. 2011;7:419–25. 21389777 10.4161/hv.7.4.14120 PMC3360071

[9] Shati AA Al-Qahtani SM Alsabaani AA. Perceptions of parents towards COVID-19 vaccination in children, Aseer region, Southwestern Saudi Arabia. Vaccines. 2022;10:1222. 36016110 10.3390/vaccines10081222 PMC9414894

[10] Alhazmi A Alamer E Daws D. Evaluation of side effects associated with COVID-19 vaccines in Saudi Arabia. Vaccines. 2021;9:674. 34207394 10.3390/vaccines9060674 PMC8235009

[11] Boivin Z Martin J. Untimely myocardial infarction or COVID-19 vaccine side effect. Cureus. 2021;13:e13651. 33824804 10.7759/cureus.13651 PMC8012173

[12] Cirillo N. Reported orofacial adverse effects of COVID-19 vaccines: the knowns and the unknowns. J Oral Pathol Med. 2021;50:424–7. 33527524 10.1111/jop.13165 PMC8013400

[13] Bogdanov G Bogdanov I Kazandjieva J Tsankov N. Cutaneous adverse effects of the available COVID-19 vaccines. Clin Dermatol. 2021;39:523–31. 34518015 10.1016/j.clindermatol.2021.04.001 PMC8076732

[14] Morgan E. Pandemic periods: why women’s menstrual cycles have gone haywire. The Guardian. 2021:25. https://www.theguardian.com/society/2021/mar/25/pandemic-periods-why-womens-menstrual-cycles-have-gone-haywire. [cited 2, August 2023].

[15] Lebar V Laganà AS Chiantera V Kunič T Lukanović D. The effect of COVID-19 on the menstrual cycle: a systematic review. J Clin Med. 2022;11:3800. 35807090 10.3390/jcm11133800 PMC9267255

[16] King EM Albert AY Murray MCM. HIV and amenorrhea: a meta-analysis. AIDS. 2019;33:483–91. 30531313 10.1097/QAD.0000000000002084

[17] Rostami Dovom M Ramezani Tehrani F Djalalinia S Cheraghi L Behboudi Gandavani S Azizi F. Menstrual cycle irregularity and metabolic disorders: a population-based prospective study. PLoS One. 2016;11:e0168402. 27992506 10.1371/journal.pone.0168402 PMC5161370

[18] Alvergne A Kountourides G Argentieri MA. COVID-19 vaccination and menstrual cycle changes: a United Kingdom (UK) retrospective case-control study. iScience. 2023;26:106401. 36987520 10.1016/j.isci.2023.106401 PMC10015085

[19] Bouchard TP Schneider M Schmidt M Manhart M Fehring RJ. Menstrual cycle parameters are not significantly different after COVID-19 vaccination. J Women Health (Larchmt). 2022;31:1097–102. 10.1089/jwh.2022.0097 35723654

[20] Edelman A Boniface ER Benhar E. Association between menstrual cycle length and coronavirus disease 2019 (COVID-19) vaccination: a US Cohort. Obstet Gynecol. 2022;139:481–9. 34991109 10.1097/AOG.0000000000004695 PMC8936155

[21] Alvergne A Von Woon EV Male V. Effect of COVID-19 vaccination on the timing and flow of menstrual periods in two cohorts. Front Reprod Health. 2022;4:952976. 36303656 10.3389/frph.2022.952976 PMC9580734

[22] Male V. Menstrual changes after Covid-19 vaccination. BMJ. 2021;374:n2211. 34526310 10.1136/bmj.n2211

[23] Al-Mehaisen LMM Mahfouz IA Khamaiseh K Al-Beitawe SN Al-Kuran OAH. Short term effect of corona virus diseases vaccine on the menstrual cycles. Int J Womens Health. 2022;14:1385–94. 36164386 10.2147/IJWH.S376950 PMC9507976

[24] Sharp GC Fraser A Sawyer G. The COVID-19 pandemic and the menstrual cycle: research gaps and opportunities. Int J Epidemiol. 2022;51:691–700. 34865021 10.1093/ije/dyab239 PMC8690231

[25] Suzuki S Hosono A. No association between HPV vaccine and reported post-vaccination symptoms in Japanese young women: results of the Nagoya study. Papillomavirus Res. 2018;5:96–103. 29481964 10.1016/j.pvr.2018.02.002 PMC5887012

[26] Teijaro JR Farber DL. COVID-19 vaccines: modes of immune activation and future challenges. Nat Rev Immunol. 2021;21:195–7. 33674759 10.1038/s41577-021-00526-x PMC7934118

[27] Laganà AS Veronesi G Ghezzi F. Evaluation of menstrual irregularities after COVID-19 vaccination: results of the MECOVAC survey. Open Med (Wars). 2022;17:475–84. 35350834 10.1515/med-2022-0452 PMC8919838

[28] Chourasia UH Khormi AH Jawkhab HA. Determining the effect of COVID-19 on the menstrual cycle among women of reproductive age group in the Jazan region: a cross-sectional study. Cureus. 2022;14:e32431. 36644095 10.7759/cureus.32431 PMC9832938

[29] Centers For Disease Control And Prevention. National Center for Immunization and Respiratory Diseases (NCIRD), division of viral diseases; 2022. COVID-19 vaccines for people who would like to have a baby. https://www.cdc.gov/covid/vaccines/pregnant-or-breastfeeding.html. [cited February 19, 2025].

[30] Alghamdi AN Alotaibi MI Alqahtani AS Al Aboud D Abdel-Moneim AS. BNT162b2 and ChAdOx1 SARS-CoV-2 post-vaccination side-effects among Saudi vaccines. Front Med (Lausanne). 2021;8:760047. 34692740 10.3389/fmed.2021.760047 PMC8531069

